# Has the COVID-19 pandemic affected nursing students’ career self-efficacy and professional calling? The mediating impact of professional identity

**DOI:** 10.1186/s12909-022-03833-6

**Published:** 2022-11-05

**Authors:** Li Yang, Mengfan Xu, Jinke Kuang, Kexin Zhou, Xuemei Zhu, Lingna Kong, Li QI, Heng Liu

**Affiliations:** 1 School of Nursing, Qingdao University, 15 Ningde Road, Qing Dao, Shandong Province 266071 P.R. China; 2 School of Nursing, The Second Affiliated Hospital of Harbin Medical University, Harbin Medical University, 246 Xuefu Road, Harbin, Heilongjiang Province 150081 P.R. China; 3 School of Nursing, Chongqing Medical University, No. 1 Youyi Road, Chongqing, 400016 P.R. China; 4 School of Nursing, Qiqihaer Medical University, 333# Bukui North Road, Qiqihar, Heilongjiang Province 161006 P.R. China

**Keywords:** Professional calling, COVID-19, Professional identity, Career self-efficacy, Nursing students

## Abstract

**Background:**

Professional calling (PC) is crucial for ascertaining their professional goals and fulfilling career choices in nursing students. Thus, understanding its antecedents and helping schools improve PC among nursing students is critical. This study aims to explore whether professional identity (PI), as a crucial antecedent of PC, acts as an intermediary between career self-efficacy (CSE) and professional calling during the COVID-19 pandemic.

**Methods:**

A sample of 565 nursing students were selected by a web-based survey through convenience sampling. The study was conducted from October to November 2020. Measures of CSE, PI, and PC were assessed during the COVID-19 pandemic. We analyzed demographic data and the correlation of the research variables. The significance of the mediation effect was assessed using a bootstrap method with SPSS.

**Results:**

CSE during the COVID-19 pandemic outbreak (*r* = 0. 359, *p* < 0. 01) and PI (*r* = 0. 670, *p* < 0. 01) were both relevant to PC among nursing students. In addition, CSE had a positive indirect effect on PC through PI (β = 0. 288, *p* < 0. 05).

**Conclusions:**

Higher scores in CSE and a better PI were associated with PC in nursing students. Furthermore, a better CSE had an indirect effect on the PC of students through PI. The favorable evidence in our study confirms that nursing educators can adopt PI interventions to improve the sense of PC among nursing students.

## Background

Due to its infectious nature and serious complications to a generally susceptible population, the COVID-19 pandemic posed a huge threat to human health [1, 2]. Nurses play an important role in health systems’ response to the COVID-19 pandemic as they are the frontline main healthcare professionals (HCPs) directly involved in the treatment and care of patients [3–5]. Driven by professional calling (PC), nurses have taken the initiative in fighting the pandemic although they are extremely vulnerable to the threat of COVID-19 [6]. PC refers to individual’s passion and power to gain responsibility and realize their life value in a particular profession [7]. Nurses with high PC will instinctively invest more time in their work and better shoulder the heavy task of saving the dying and healing the wounded [8]. As the reserve force for future nurses, nursing students’ PC is crucial for ascertaining their professional goals and the fulfillment of their career choice [6, 9]. The higher the PC of nursing students, the more can they accept the nursing profession [9] and improve nursing competence. Moreover, PC is a key factor for the cognition of the nursing profession, the establishment of career goals [10], fulfillment of career choice [11], and enhancement of the willingness to shoulder social responsibilities for nursing students. PC is necessary to cultivate qualified nursing students for the nursing profession. In addition, the conceptualization of PC has driven the development of the nursing profession. Since its introduction, PC as a concept has continued to gain significance and capture the attention of researchers in many fields [12–14]. Many believe that individuals' perceptions of their PC are shaped by the environment [15]. Therefore, it is unsurprising that COVID-19 may be affecting nursing students’ PC. Nursing students’ participation in clinical practice during COVID-19 has not only been affected by scarce personal protective equipment but also by the risks to patients and staff [16]. Therefore, nursing schools have moved to online learning, often suspending clinical practice [17], which is affecting important experiences required as part of nursing education [18]. This situation may consequently also impact their PC [10] and increase job burnout and turnover rate [19]. Given these potential negative results, affirming the antecedents of PC and helping schools improve such PC among nursing students is crucial to designing effective and targeted interventions.

Career self-efficacy (CSE) is an important antecedent variable affecting PC of nursing students. As a critical challenge to the current healthcare system, COVID-19 has led to many disruptions in nursing education [18, 20]. These disruptions have had several effects on the mindsets of nursing students, such as on their CSE [21–23]. CSE refers to the confidence in oneself to perform a variety of tasks and activities related to one's career [24]. During the COVID-19 pandemic, the CSE of nursing students may be affecting their belief or confidence in their capacity to complete nursing activities [25].

Recent research on nursing students has confirmed that COVID-19 has been influencing their professional identity (PI) [26–28]. PI is defined as a clear understanding of one’s professional values and ethics, interests, abilities, and goals, along with the structure that links such self-knowledge to one's professional role [29]. Similar to the impact of the SARS epidemic in 2003 [30], COVID-19 has enhanced nursing students’ PI, reflecting how a public health emergency influences such identity among students [31]. Research has established a significant correlation between CSE and PI among nursing students [21, 22, 32]. Moreover, a recent study during the COVID-19 pandemic in China has indicated that CSE may be an important factor influencing PI among first-year nursing students [33]. Nursing students with a high level of CSE are confident in their future career development. Therefore, they are more emotionally invested in their profession and have a higher sense of PI.

PI is not only regarded as an outcome variable predictor, but also as a predictor of internal factors, such as PC. It is considered a major antecedent of PC and also connected with one’s career choices [16]. These two constructs are theoretically related, as PC entails a sense of identification with the domain of one’s profession [8]. Empirical research has confirmed this close correlation among pharmacy, dentistry, and medicine students. Moreover, a longitudinal study of PI and PC in students demonstrated that both PI and PC decrease over time along with their strong correlation [34].

Recent studies on different populations have also discovered that students’ PC positively correlated with CSE [34–36].The Chinese scholar found that the higher the level of CSE, the more confident nursing students accept challenges and improve their PC [37]. A survey of 855 undergraduate students from three universities in Atlantic Canada highlighted that one's PC is moderately linked to one's CSE [35]. More importantly, other research has demonstrated that one’s PC to medicine can strengthen a student’s CSE [35]. Goodin [38] noted a similar result, reporting that PC is particularly important in developing career commitment among students with low CSE.

Based on the above discussion, we proposed the following hypotheses:

- Hypothesis 1. The CSE should be directly related to PC. Higher CSE scores of nursing students should be related to a stronger sense of PC.
- Hypothesis 2. CSE is directly related to PI. The higher the CSE score, the higher the PI should be.
- Hypothesis 3. A higher CSE score should relate to better PC indirectly through stronger PI. PI should play a mediating role between CSE and PC.

## Methods

### Study design

This is a questionnaire-based, anonymous cross-sectional study. The study aims to explore whether the PI plays a mediation effect between CSE and PC in nursing students during the COVID-19 pandemic.

### Sample

The appropriate sample size was estimated based on a calculation of 5–10 times the number of research items [39]. There were 54 items measured in our study. Considering 5% invalid questionnaires, our valid sample size was at least 284.

### Data collection

Due to the outbreak of COVID-19, a web-based online survey was conducted to reduce the risk of infection from face-to-face contact. The questionnaire was entered into electronically the Chinese Star platform (website: https://www.wjx.cn). Participants answered questionnaires by clicking on a Chinese Star platform link on their phone or computer. In our research group, 8 students were selected to pretest the questionnaire through face-to-face interviews, who can make suggestions and points they do not understand. The final questionnaire was modified based on their feedback to ensure its comprehensibility and preciseness. And the final questionnaire includes the purpose and content of the study, demographic characteristics, the career self-efficacy scale for nursing students during the COVID-19 pandemic, the professional identity scale, and the Chinese calling scale. Each participant filled out only one questionnaire using a single social media account and IP address. The platform does not allow submission if the questionnaire is incomplete. In addition, the platform can mark the questionnaire response time. The average time spent filling out the questionnaire during the pretest was 5 min. Those whose answers took less than five minutes were flagged as failing, which is also a method of questionnaire quality control. Moreover, we set the reverse questions. These settings ensure the authenticity and validity of the questionnaire.

### Participants

For the sake of the representativeness, our research group contacted nursing teachers from nursing schools we knew from 18 provinces across the country. Then, they forwarded the questionnaire link to nursing students who met the inclusion criteria through the WeChat network. Our inclusion criterion was that the individual be a full-time nursing student. The voluntary nature of student participation and the informed consent statement was included in the questionnaire, wherein it was stated that completing the investigation implied consent to participate. A total of 655 nursing students agreed to participate in our survey from October to November 2020.

### Variable measurements

#### Career self-efficacy

The CSE scale had been developed to measure the CSE of nursing students during the COVID-19 pandemic outbreak. In this study, CSE refers to the choice and commitment of nursing students to the nursing profession during the COVID-19 pandemic outbreak. To evaluate the validity of the scale content, the scale was reviewed by seven experts in the fields of nursing education, psychology, and public health. Each of these experts has more than a decade of experience in their respective fields. They assessed each item on a four-point Likert scale (4 = "very relevant", 3 = "relevant", 2 = "irrelevant", and 1 = "very irrelevant"). To ensure consistency among experts, the item-level Content Validity Index (I-CVI) was calculated. I-CVI was calculated as the response ratio of 3 and 4 points for each item, and I-CVI of 0.78 or higher is considered appropriate [40]. The I-CVI values of the five items all met the standard of 0.88 to 1, and the mean value of scale-level CVI (S-CVI) was 0.91. The final CSE scale includes five items: (1) “COVID-19 has given me a new understanding of the future nursing profession,” (2) “COVID-19 has had a positive effect on my nursing profession,” (3) “COVID-19 has made me realize that the nursing occupation is sacred,” (4) “COVID-19 has enhanced my responsibility and calling in the nursing profession,” and (5) “COVID-19 has strengthened my determination to pursue the nursing profession.” Each item was scored on a three-point Likert-type scale, with a total score of 15. A high score indicated a high-level of CSE. Before the formal investigation, a pilot testing was conducted with 36 nursing students to examine the reliability of the items. The Cronbach’s alpha for the CSE scale in the pilot testing was 0.816. In this study, Cronbach’s alpha for the CSE scale was 0.829, and the half reliability was 0.814.

### Professional identity

PI was assessed using the Professional Identity Scale developed by a Chinese researcher [ 41]. The scale consisted of 38 items that measured six dimensions: professional cognition, professional emotion, professional commitment, professional behavior, professional expectations, and professional values. Each item was scored on a five-point Likert-type scale, from one “absolutely inconsistent” to five “absolutely consistent,” with a total score of 190. High scores indicated high levels of PI. Cronbach’s alpha for the Professional Identity Scale in the original study [41] and in this study were 0.908 and 0.954, respectively. The half reliability for the Professional Identity Scale in the original study [41] and in this study were 0.929 and 0.825, respectively.

### Professional calling

PC was assessed using the Chinese calling scale (CCS) developed by the Chinese researcher Zhang et al. [42]. The CCS comprises 11 items that measured three dimensions: value and significance, guiding force, and altruistic contribution. Each item was scored on a five-point Likert-type scale, from one “absolutely inconsistent” to five “absolutely consistent,” with the highest score of 55. High scores indicated higher levels of PC. Based on previous studies of Chinese nursing students, this scale indicated better internal consistency [43, 44]. When the Chinese calling scale was originally developed, Cronbach’s alpha was 0.890 and half reliability was 0.929 [42]. In this study, Cronbach’s alpha was 0.879 and half reliability was 0.916.

### Ethical considerations

This study protocol accorded with the Helsinki Declaration and was authorized by the Medical Ethics Committee of Affiliated Hospital of Qingdao University (No.QYFYWZLL25900). The authors offered a short explanation of the study to potential participants via the internet, who were also assured that participation was completely voluntary. Nursing students who finished the investigation were regarded as agreeing to participate in this study. To ensure anonymity and confidentiality, we did not collect participants’ names and other identification information. The collected data was stored in a password-protected Chinese Star platform that only the author can access. The data was removed after analysis. The participants could not obtain any award after they answered our electronic survey.

### Data analysis

If the questionnaire is not completed, the platform does not allow submission. Therefore, no data were missing in this study. Out of the 655 questionnaires received, 90 invalid questionnaires were deleted. Invalid questionnaires included those which had a response time of fewer than five minutes and provided contradictory answers to the reverse questions. The final effective participants were 565, and the final questionnaire response ratio was 86.3%. The normality test of the data was conducted using the Kolmogorov–Smirnov test. We conducted a descriptive analysis of our demographic data and correlations among the study variables. In addition, Harman’s single-factor test was conducted to confirm common method variance was not a threat in our data set [45]. In our mediation analysis, our independent variable was CSE, the mediator was PI, and the dependent variable was PC. We calculated their interrelationships and used the PROCESS macro model 4 in SPSS for the mediation model. Bootstrapping was adopted to measure the indirect effect. We also applied a 95% confidence interval. When the confidence interval (CI) contains zero, there is no significant mediating effect (indirect) effect. We used 5000 bootstrap samples. The analysis was conducted using SPSS 25. 0 for Windows.

## Results

### Participant demographics

Of the 565 nursing students who completed the survey, there were 55 male students (9.70%) and 510 female students (90.30%). Their average age was 20.94 years (SD = 1.98). Among them, the proportion of lower grade, middle grade and high grade respectively was 26.00%, 49.60% and 24.40%. More than half of nursing students had no clinical practice experience. The overall mean scores of PI and PC were 126.49 (SD = 19.38) (medium level), 40.16 (SD = 6.40) (medium level) respectively. 69% of nursing students admitted that COVID-19 had a positive impact on their profession.

### Preliminary and descriptive results

Results of Harman’s single-factor test revealed that the variance explanation rate of the unrotated first common factor in principal component analysis was 36.04% (less than 40%), which indicates that common method variance is not a serious deficiency in this study [45]. The Pearson correlations of the study variables are presented in Table 1. The results show that PC had a significant correlation with CSE (*r* = 0. 359, *P* < 0.01) and PI (*r* = 0. 670, *P* < 0.01). PI also had a significant correlation with CSE (*r* = 0. 479, *P* < 0.01).

**Table 1:** Correlations among  career self-efficacy, professional identity and professional calling

	1	2	3
1. Career self-efficacy	1		
2. Professional identity	0. 479**	1	
3. Professional calling	0. 359**	0. 670**	1

^**^P < 0. 01

### The mediating effect of PI in the association between CSE and PC

**Fig. 1:**
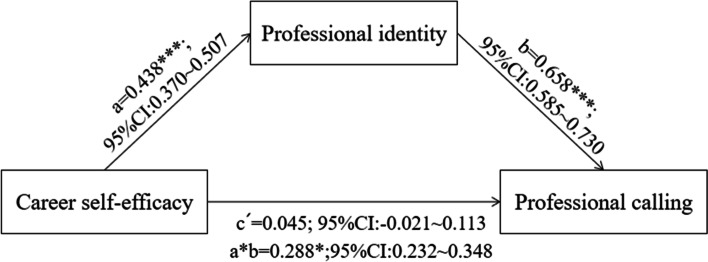
Mediating role of professional identity in the relationship between career self-efficacy and professional calling, with regression coefficients, indirect effects, and bootstrapped CI. *p < 0. 05,**p < 0.01,***p < 0. 001; CI = confidence interval. a = path from independent variable to mediating variable. b = path from mediating variable to dependent variable. c' = direct effect of independent variable on dependent variable. a*b = indirect effect of independent variable on dependent variable through the mediating variable

The sociodemographic variables (age, gender, grade, clinical practice experience) were controlled for, before identifying the significance of the direct, indirect, and total effects in the mediation model. The results show that the direct effect of CSE on PC is not statistically significant at 0.045 (95% confidence interval [-0.021–0.113]), as the confidence interval of this direct effect includes zero. In addition, the indirect effect of PI is statistically significant at 0.288. Therefore, the results show that PI acted as a complete mediating role between CSE and PC. The proportion of mediation of PI was 86.04%. The model is presented visually in Fig. 1.

## Discussion

In this study, more than half of nursing students admitted that COVID-19 had a positive impact on their profession, which was consistent with a previous study [26]. The nurses who volunteer on the front lines, managing the pandemic, reflect a professional image that is widely appreciated by the public. This situation can encourage a positive professional view among nursing students. However, compared with the data gathered prior to the COVID-19 pandemic using similar research tools and study populations [10], the current study examined the reduced PC in nursing students during the pandemic. Major emergencies affect the perspective of students on the meaning of work and life, thereby damaging the impulse to seek out meaningful pursuits [34]. The touching stories of nurses during the COVID-19 pandemic, which have drawn unprecedented public attention, have made nursing students aware of the professional risk [46, 47] and face the choice between their duties and the risk of contracting the virus in their future clinical work [48]. The pandemic has led to the suspension of clinical practice and face to face teaching, and forced nursing students to turn to online learning [49]. Therefore, nursing students can not experience professional value and sense of being needed in practice [50]. More importantly, nursing students are affected by the nature of high-risk work. The COVID-19 pandemic has undoubtedly intensified these negative experiences, thus deepening these negative effects [20].

First, our correlation analysis showed that PC has been positively and significantly associated with both CSE and PI during the COVID-19 pandemic. This finding agrees with that in previous studies as well as with social cognitive career theory (SCCT). This theory points out that person–environment interactions produce learning experiences, affect one's CSE, and shape one's career. In addition, the environment has a direct influence on career behavior [51]. In interviews with medical interns, it was found that PC was formed by both the recognition of one's ability, and one's external environment [52].

Second, we surveyed the complete mediating role of PI in the relationship between CSE and PC. Although existing research on PI suggests that CSE is positively related to PC, this relationship was not treated in the context of COVID-19. Our results show that PI mediates the relationship between CSE and PC, thus providing full support for our third hypothesis. Higher CSE promotes more PI and, in turn, greater PC. The higher the CSE, the more confidence a student has in one's career. Nursing students with high CSE are more motivated [53]; hence, they are able to understand and perform nursing practical tasks [54] and consequently have higher levels of PI. In addition, existing literature shows that PI can be considered as one of the correlates of PC [55, 56]. When nursing students have high level of PI, they tend to identify with the meaning and value of nursing and have a higher PC [23]. However, its mediating role on the relationship between CSE and PC was not identified in the existing literature [21]. Therefore, our findings extend the literature on CSE, PI. and PC through specifying the role of PI as a mediator in the CSE and PC relationship.

### Implications

Consistent with previous studies [ 41, 57], our results indicated that the PC and PI of nursing students were at medium level. And there's a lot of room for improvement. Moreover, the complete mediating role of PI between CSE and PC further confirms that adopting PI interventions can contribute to PC career development. Clinical practice is the key period for nursing students to form PI [28, 58]. However, the suspension of clinical practice during the COVID-19 pandemic has affected the PI of nursing students [28]. Online simulation training and volunteer activities can make up for the lack of clinical practice to a certain extent [22]. A previous study identified that the low social status and attractiveness of nursing are major barriers to develop PI in nursing students [59]. The media portrays nurses as respectable, self-sacrificing heroes [31]. Therefore, social media and online platforms should be fully utilized in order to further contribute to the establishment of positive nursing image. The clearer the nursing students' self-awareness, the higher their PI is [60]. Strategies to promote the self-concept of nursing are crucial to increase the students’ recognition of nurses as professionals [31]. Hence, we should guide nursing students to fully reflect and understand the nursing profession. Nursing students with high resilience may be less likely to perceive the negative effects of the COVID-19 pandemic [48, 61] and more likely to develop positive perceptions and evaluations of the nursing profession, hereby enhancing PI [62]. Therefore, it is necessary to integrate resilience training into nursing humanistic curricula [63, 64]. And among nursing students, resilience training can be achieved through the support of influencers [61]. Nursing students' professional identities are shaped by exposure to role models in the profession. HCPs who are fighting on the front lines for pandemic prevention are strong role models, and nursing educational institutions can invite first-line nurses to share their experiences in responding to healthcare crisis [22]. The stronger the students’ CSE is, the higher their PI is. Therefore, strengthening the cultivation of innovative behavior of nursing students [65] and helping them understand more professional information [66] can help improve their PI.

### Limitations and recommendations

This study has several limitations. First, the cross-sectional nature of this research also hindered the examination of causal relationships between variables. Second, due to the characteristics of the nursing profession itself there were a large number of female nursing students compared to males, which may underrepresent male nursing students. Third, all data collected were through self-reported questionnaires, which inevitably creates response bias and social desirability bias. In addition, although the professional identity scale we used was validated, not specifically for nursing students, which is a limitation of this study. Finally, the study was conducted in one country and may not be representative of nursing students in other countries. Moreover, it used a convenience sampling method. The above situation may limit the generalization of our findings.

Given these limitations, longitudinal studies are needed to further provide more reliable evidence in the future. The study should be conducted in the future with more nursing students in different countries, and probability sampling should be used to make the sample representative. Moreover, a qualitative study would have been more informative to support the quantitative findings.

## Conclusions

In our study, PI played a complete mediating role between CSE and PC during the COVID-19 pandemic. Hence, hospitals and nursing institutions should consider how to design measures that enhance PI among nursing students to boost their sense of PC. Ultimately, by improving their PC, we can ensure that the quality and advancement of nursing services continue. Nursing educators should help students understand the epidemic and incorporate relevant findings into educational activities that may enhance nursing students' professional identity.

## Data Availability

The datasets generated and/or analysed during the current study are not publicly available due but are available from the corresponding author on reasonable request.

## Abbreviations

PI: Professional Identity
CSE: Career Self-efficacy
PC: Professional Calling
HCPs: Healthcare professionals
